# Acromegaly in an elderly male with hepatic malignancy: a case report of a diagnostic dilemma

**DOI:** 10.1097/MS9.0000000000004375

**Published:** 2025-11-24

**Authors:** Rakesh Kumar Sah, Deekshya Devkota, Chandan Kumar Sah, Anil Pathak

**Affiliations:** a Department of Internal Medicine, Shree Birendra Hospital, Kathmandu, Nepal; b Nepalese Army Institute of Health Sciences, Kathmandu, Nepal

**Keywords:** acromegaly, case report, hepatic malignancy, IGF-1, paraneoplastic syndrome, pituitary lesion

## Abstract

**Introduction and importance:**

Acromegaly is usually caused by a growth hormone (GH)-secreting pituitary adenoma; nonpituitary causes are rare, especially in elderly patients. Paraneoplastic insulin-like growth factor-1 (IGF-1) excess from hepatic malignancy is quite uncommon, and its coexistence with a pituitary infundibular lesion presents a diagnostic challenge.

**Case Presentation:**

A 78-year-old diabetic man presented with coarse facial features, macroglossia, and increased interdental spacing. IGF-1 was elevated (218.6 ng/mL) with normal GH, ACTH, and prolactin. The 8 AM serum cortisol was high (36.27 μg/dL), suggesting ACTH-independent hypercortisolism. MRI brain revealed an enhancing pituitary infundibular lesion (pituicytoma vs granular cell tumor). CECT A + P exhibited a large hepatic mass with pulmonary metastases, elevated tumor markers (CEA, CA 19-9, AFP), and normal adrenal glands. Findings favored paraneoplastic IGF-1 excess and hypercortisolism from hepatic malignancy, with the pituitary lesion likely incidental. The family declined further workup, and the patient was lost to follow-up.

**Clinical discussion:**

Hepatic malignancy can rarely produce IGF-1 or cortisol-like substances, leading to acromegaly-like features and hypercortisolism independent of pituitary GH or ACTH secretion. The coexistence of paraneoplastic IGF-1 excess, ACTH-independent hypercortisolism, and an incidental pituitary lesion has not been previously reported. This case highlights the need to consider nonpituitary causes of acromegaly, especially in elderly patients with atypical biochemical or imaging findings.

**Conclusion:**

Non-pituitary causes of acromegaly should be considered in elderly patients with atypical biochemical profiles, as hepatic malignancy can rarely produce paraneoplastic IGF-1 excess even in the presence of incidental pituitary lesions.

## Introduction

Acromegaly is an endocrine disorder resulting from a dysregulated hypersecretion of growth hormone and its target hormone, insulin-like growth factor 1 (IGF-1), usually caused by a growth hormone-secreting pituitary adenoma^[1]^. Acromegaly is most commonly diagnosed in adults between 35 and 45 years of age, with new-onset cases in elderly individuals (≥ 65 years) being exceptionally rare^[2]^. Diagnosis is often delayed by 5–10 years due to insidious onset and slow progression^[3]^.

Growth hormone secreting pituitary adenomas are the cause of acromegaly in over 99% of patients^[4]^. In contrast, instances linked to nonpituitary malignancies are exceedingly uncommon. Among these rare exceptions, hepatic malignancies have occasionally been linked to ectopic insulin-like growth factor-1 (IGF-1) production, resulting in acromegaly-like clinical features; however, such reports remain exceedingly uncommon.

**HIGHLIGHTS**

- This case presents an elderly patient with hepatic malignancy and pulmonary metastases presenting with paraneoplastic acromegaloid features (elevated IGF-1 with normal GH) and hypercortisolism, alongside an incidental pituitary infundibular lesion.
- Coexistence of hepatic malignancy and pituitary lesion complicated differentiation between primary pituitary and paraneoplastic causes of acromegaly
- To the best of our knowledge, this is the first reported case of a hepatic malignancy causing paraneoplastic IGF-1 excess and hypercortisolism in a patient who also had a pituitary infundibular lesion.

Here, we present a rare and diagnostically challenging case of an elderly male with clinical and biochemical features of acromegaly in the setting of hepatic malignancy, with no evidence of pituitary adenoma. To the best of our knowledge and research, such cases have rarely been reported. This case contributes to the limited literature on paraneoplastic endocrine manifestations of acromegaly associated with hepatic malignancy and highlights the importance of maintaining a high index of suspicion for nonpituitary causes of acromegaly in elderly individuals.

This case report adheres to the CARE guidelines^[5]^, is compliant to the TITAN guidelines 2025^[6]^ and presents the patient’s clinical presentation, investigations, and management.

## Case presentation

**Figure 1.**
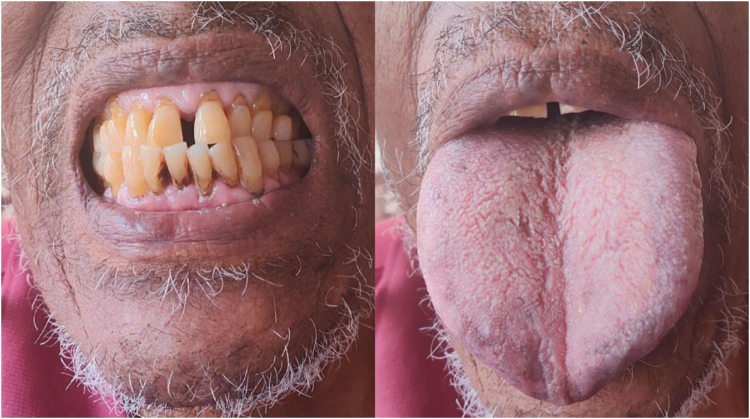
Increased interdental spacing and macroglossia in our patient with acromegaly.

**Figure 2.**
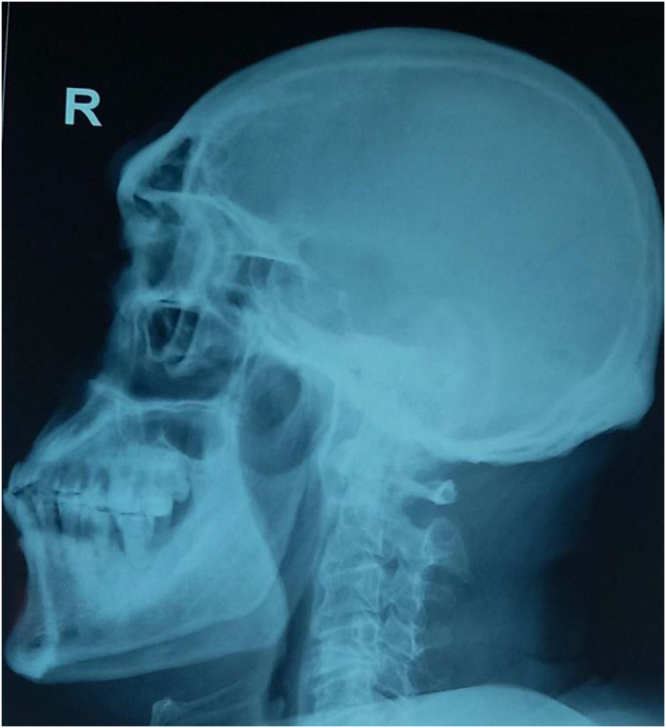
X-ray head lateral view showing a prominent forehead and jaw.

A 78-year-old male, a known case of type 2 diabetes mellitus on Tab. Glimepiride for the past 6 years, presented to the emergency department with complaints of loss of consciousness lasting 30 minutes, dizziness for 1 week, fever for 1 week, and lower urinary tract symptoms for 4 months. On examination, he appeared ill-looking with bilateral pitting pedal edema. Chest auscultation revealed crepitations over the right infra-axillary region. He was admitted with a diagnosis of severe symptomatic hypoglycemia secondary to sulfonylurea and decreased intake, right lower zone pneumonia, and suspected acromegaly. Acromegaly was suspected based on his coarse facial features, including a prominent forehead, protruding jaw, increased interdental spacing, macroglossia, and thick and rough skin (Figures 1 and 2). His arm span was 166.4 cm, whereas his height was 165.1 cm, suggesting a slightly increased arm span to height ratio. Routine laboratory investigations revealed decreased random blood sugar, an elevated total leukocyte count with lymphocytic predominance, an increased platelet count, and a reduced hemoglobin level. His fasting lipid profile, thyroid function test, and serum electrolyte levels were within normal limits (Table 1).

**Table 1:** The patient’s laboratory workup

Parameter	Value	Reference range
WBC	15.2 × 10^3^/µL [*N* = 82%]	(4–11) × 10^3^/µl
Hb	10.22 g/dL	(13–17) g/dL
PLT	504 × 10^3^/µl	(150–450) × 10^3^/µl
Serology	Non-reactive	
Total bilirubin	0.85 mg/dL	0.3–1.2 mg/dL
Direct bilirubin	0.28 mg/dL	0–0.2 mg/dL
ALT	15.20 U/L	13–40 U/L
AST	52.46 U/L	13–40 U/L
ALP	136.03 U/L	42–128 U/L
Urea	25.02 mg/dL	13–43 mg/dL
Creatinine	1.23 mg/dL	0.6–1.3 mg/dL
RBS	13.84 mg/dL	70–140 mg/dL
FBS	100.2 mg/dL	70–110 mg/dL
PPBS	142.6 mg/dL	110–140 mg/dL
HBA1C	6.1%	4–5.7%
CEA	121 ng/mL	<3.8 ng/mL
AFP	1000 IU/mL	<5.8 IU/mL
CA 19-9	154.3 U/mL	<39 U/mL
Human growth hormone	1.82 ng/mL	0.020–3.893 ng/mL
IGF-1	218.6 ng/mL	59–181 ng/mL
ACTH	19.80 pg/mL	4.70–48.80 pg/mL
Serum cortisol [8 AM]	36.27 µg/dL	8.7–22.4 µg/dL
Serum prolactin	8.16 ng/mL	2.6–13.1 ng/mL

ACTH, adrenocorticotropic hormone; AFP, alpha fetoprotein; ALP, alkaline phosphatase; ALT, alkaline aminotransferase; AST, aspartate aminotransferase; CA 19-9, IGF-1, insulin-like gGrowth factor 1; CBC, complete blood count; CEA, carcinoembryonic antigen; FBS, fasting blood sugar; Hb, hemoglobin; PPBS, post-prandial blood sugar; PLT, platelets; PT/INR, Prothrombin time/International normalized ratio; RBS, Rrndom blood sugar; WBC, white blood cells.

Ultrasound of the abdomen and pelvis demonstrated a heterogeneously hypoechoic, lobulated solid lesion in the right lobe of the liver, measuring 9.4 × 7.3 cm, with minimal vascularity, suggesting a neoplastic etiology. Additionally, a simple hepatic cyst was noted in the left lobe of the liver along with a cortical cyst in the left kidney.

**Figure 3.**
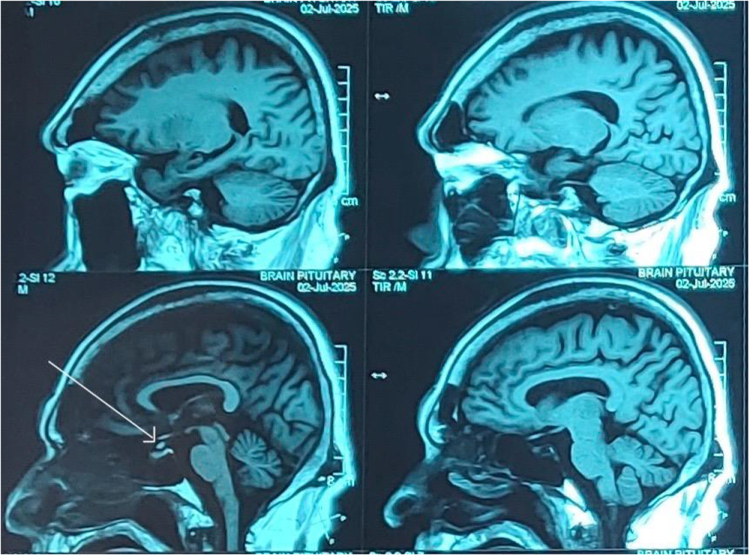
Postgadolinium enhancement MRI brain showing abnormal enhancing thickening of the pituitary infundibulum (white arrow) with no evidence of macroadenoma or microadenoma.

As part of the acromegaly workup, a contrast-enhanced MRI of the brain was performed, which revealed an abnormal enhancing thickening of the pituitary infundibulum with post-gadolinium enhancement and no evidence of macroadenoma or microadenoma. Differential diagnoses included pituicytoma of the infundibulum and pituitary granular cell tumor (Fig. 3). Tissue diagnosis was recommended; however, it was not pursued as the patient’s party denied further workup.

**Figure 4.**
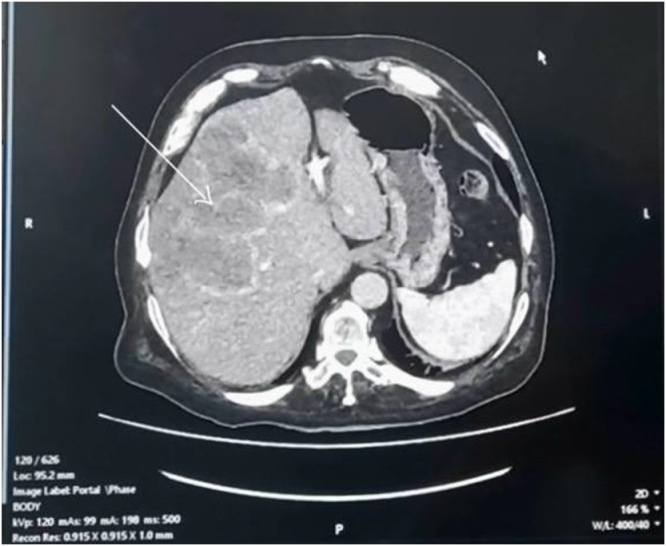
Axial view of CECT A + P showing a lobulated lesion in the liver measuring 16.1 × 13.7 × 15.3 cm with an enhancing capsule (white arrow).

Further workup revealed a normal human growth hormone level with an elevated IGF-1 level. Tumor markers CEA, CA 19-9, and AFP were also elevated. Contrast-enhanced CT scan of the abdomen and pelvis showed a lobulated lesion in the liver measuring 16.1 × 13.7 × 15.3 cm (AP × TR × CC) with an enhancing capsule, along with multiple enhancing subpleural-based nodules in both lung parenchyma – suggestive of a likely neoplastic liver lesion with pulmonary metastases (Fig. 4). Although tissue diagnosis was advised in this case, the family declined further diagnostic workup, which impeded definitive management.

His ACTH and prolactin levels were within normal limits, while the 8 AM serum cortisol level was elevated. The patient’s outcome could not be determined as he was lost to follow-up.

## Discussion

Acromegaly is a rare disease overall, and data on elderly patients with acromegaly remain limited^[7]^. Clinical manifestations range from coarse facial features – such as a prominent forehead, prognathism, wide dental spacing, macroglossia, thickened skin, and skin tags – to involvement of the cardiovascular, respiratory, endocrine, metabolic, and musculoskeletal systems^[8]^. Baseline biochemical parameters for the diagnosis of acromegaly include the measurement of fasting or random GH and of IGF-I^[9]^. The diagnosis is often delayed due to the indolent and insidious nature of the disease. However, untreated acromegaly is associated with significant morbidity and reduced life expectancy. Adenomas causing acromegaly are typically larger than 1 cm at the time of diagnosis and are almost always visible on conventional MRI. Therefore, if pituitary MRI is unremarkable, a contrast-enhanced CT scan of the chest, abdomen, and pelvis should be performed to search for an ectopic source of GH or GHRH secretion^[4]^.

Acromegaly caused by a very small pituitary microadenoma that is not visualized on pituitary MRI is rare. To the best of our knowledge, only a few cases have been reported in which acromegalic patients had negative pituitary imaging, but adenomas were later identified during pituitary exploration. Doppman *et al*^[10]^ described three acromegaly patients in whom MRI imaging failed to detect a pituitary adenoma that was later discovered at surgery (resected adenoma sizes: 6, 7, and 10 mm, respectively).

Ong *et al*^[11]^ reported a case of a 76-year-old female presenting with clinical features of acromegaly, elevated IGF-1 levels, and normal growth hormone levels. Imaging studies, including MRI of the brain and CT scans of the chest, abdomen, and pelvis, were unremarkable. The patient was managed medically with octreotide and cabergoline.

However, atypical presentations without elevated GH or in the context of nonadenomatous pituitary tumors or systemic malignancy are diagnostically challenging. Our patient, an elderly male, presented with classical features of acromegaly and was found to have elevated IGF-1 but normal levels of GH, Prolactin, ACTH, and elevated serum cortisol, effectively excluding the pituitary adenoma, hypothalamic tumors secreting growth-hormone-releasing hormone (GHRH), and nonendocrine tumors causing ectopic secretion of GH or GHRH. MRI Brain in this case revealed abnormal thickening of the pituitary infundibulum with postcontrast gadolinium enhancement, with a radiological differential of pituicytoma of the infundibulum versus pituitary granular cell tumor (GCT).

Pituicytoma is a rare, slow-growing, WHO grade I tumor of the sellar and suprasellar regions, arising from the pituicytes, which are specialized glial cells in the neurohypophysis and infundibulum^[12]^. GCTs are rare neoplastic diseases of the neurohypophysis^[13]^. Neither tumor type is classically associated with hormone hypersecretion (elevated IGF and cortisol), making them unlikely as the direct source of the biochemical abnormalities or primary cause of the acromegaloid features observed in this case.

Although prolactin is best known for its reproductive and lactogenic functions, it also has extra-pituitary roles, including stimulating adrenal secretion of dehydroepiandrosterone (DHEA), cortisol, and aldosterone, and modulating the hypothalamic–pituitary–adrenal (HPA) axis during stress. Moreover, infundibular lesions commonly cause mild hyperprolactinemia through the stalk effect^[14]^; therefore, the patient’s normal prolactin level is a key finding. It not only argues against a prolactin-mediated cause of the hypercortisolism but also supports minimal or no stalk compression, reinforcing the likelihood that the pituitary lesion was an incidental finding and not responsible for the hormonal changes, while supporting a paraneoplastic origin of the hormonal changes from the hepatic malignancy.

CECT abdomen and pelvis revealed a hepatic malignancy with pulmonary metastasis and normal adrenal morphology. Markedly elevated IGF-1 with normal GH in this clinical context strongly suggests paraneoplastic acromegaloid syndrome, as hepatic tumors – particularly hepatocellular carcinoma – can ectopically produce IGF-1 or IGF-1-like peptides independent of pituitary GH regulation. The concomitant elevation of tumor markers CEA, CA 19-9, and AFP further supported the diagnosis of hepatic malignancy.

The finding of elevated cortisol with normal ACTH suggests an ACTH-independent hypercortisolism. A tumor may secrete an ACTH-like substance, such as pro-opiomelanocortin, which stimulates adrenal production of cortisol, aldosterone, and DHEA. This phenomenon has been reported in cancers like breast cancer, small cell lung cancer, carcinoid tumors, and islet cell tumors, but it hasn’t been documented in hepatocellular carcinoma (HCC). These peptides usually cause high or high-normal ACTH levels and adrenal hyperplasia^[15]^. However, in our patient, ACTH levels were normal, and DHEA and aldosterone were not measured, with no radiographic evidence of adrenal gland hyperplasia. This raises the possibility of paraneoplastic hypercortisolism originating from the hepatic malignancy itself, potentially due to tumor-secreted factors influencing cortisol metabolism. Although rare, it is a reported phenomenon in hepatic malignancy.

Sacerdote *et al*^[15]^ reported a case of a 53-year-old woman with ACTH-independent Cushing’s syndrome, characterized by markedly elevated serum cortisol and DHEA levels, but low-normal ACTH, and negative ACTH staining in the tumor. Imaging revealed metastatic HCC involving the liver, left adrenal gland, brain, and lungs, and biopsies confirmed HCC in both liver and adrenal. These findings strongly suggest ectopic cortisol and DHEA secretion by the HCC, a rare paraneoplastic phenomenon.

The primary objective in treating acromegaly is to achieve therapeutic control of GH and IGF-I secretion, as this reduces mortality to that of the general population. Treatment options include surgery, radiotherapy, and medications, with pharmacologic therapy often used postoperatively and may also serve as first-line therapy for unresectable tumors^[9]^.

Owing to the patient’s advanced age, the family opted against further diagnostic workup, thereby preventing histopathological confirmation of the malignancy and hindering definitive management. The major limitations of this case report are the lack of definitive treatment and loss to follow-up.

This case is particularly unique because of the coexistence of three rare findings:

1. A hepatic malignancy with pulmonary metastasis causing paraneoplastic IGF-1 elevation and hypercortisolism.
2. MRI evidence of an infundibular lesion with differential diagnosis of pituicytoma versus pituitary granular cell tumor.
3. Biochemical acromegaly without GH excess points towards a nonpituitary source.

This case highlights an unusual presentation of acromegaly in the absence of classical causes and emphasizes the need to consider paraneoplastic endocrine manifestations in hepatic malignancy, even when concurrent pituitary abnormalities are present. To our knowledge, no previous reports have described paraneoplastic IGF-1 excess secondary to hepatic malignancy occurring alongside a pituitary infundibular lesion, making this a unique and noteworthy contribution to the literature.

## Conclusion

This case report describes a rare presentation of paraneoplastic acromegaly caused by IGF-1 excess secondary to hepatic malignancy in an elderly patient. Normal GH levels alongside elevated IGF-1 highlight the importance of considering nonpituitary causes of acromegaly, especially in atypical cases. Increased awareness of such paraneoplastic endocrine syndromes can facilitate early recognition and improve management.

## Data Availability

Data are available on request.

## Peer and provenance statement

Not commissioned, not externally reviewed.
